# Editorial: Drug target discovery and molecular tools for parasites and host–pathogen interactions

**DOI:** 10.3389/fmolb.2026.1857697

**Published:** 2026-05-19

**Authors:** Vikky Awasthi, Namrata Anand, Surendra K. Prajapati, Yash Gupta

**Affiliations:** 1 Temple University, Philadelphia, PA, United States; 2 University of Chicago, Chicago, IL, United States; 3 The Henry M. Jackson Foundation for the Advancement of Military Medicine, Inc., Bethesda, MD, United States; 4 Department of Microbiology and Immunology, Uniformed Services University of the Health Sciences, Bethesda, MD, United States; 5 Penn State College of Medicine, Hershey, PA, United States

**Keywords:** computational biology, drug target discovery, host-pathogen interactions, multi-omics integration, translational diagnostics

The global burden of infectious diseases, particularly those caused by parasitic and bacterial pathogens, demands innovative approaches that transcend traditional reductionist methodologies. This Research Topic, “Drug Target Discovery and Molecular Tools for Parasites and Host-Pathogen Interactions,” presents a cohesive framework integrating computational prediction, experimental validation, and translational application to address complex challenges in host-pathogen biology. The ten contributions assembled here collectively demonstrate how multi-omics systems biology, artificial intelligence, rigorous statistical validation, regulatory network analysis, and field-deployable diagnostics converge to reshape our understanding of infectious disease mechanisms and therapeutic intervention strategies. Rather than presenting isolated analytical components, this Research Topic establishes an end-to-end platform supporting the entire discovery lifecycle from *in silico* hypothesis generation through experimental validation to clinical translation. The works span protozoan parasites (*Leishmania*, *Trypanosoma*, *Toxoplasma*), bacterial pathogens (*Leptospira*, *Streptococcus*), host immune modulation, structural biology, nano-enabled therapeutics, and molecular diagnostics, unified by their commitment to methodological rigor and translational relevance.

The shift from reductionist to systems-level approaches represents a fundamental paradigm change in host-pathogen research (Loiseau et al., 2020). This framework proves particularly powerful for studying parasites with unique molecular architectures, such as kinetoplastids with their polycistronic transcription and trans-splicing mechanisms (Clayton, 2019). Zhu et al. exemplify this integrative approach by dissecting exosome-mediated nucleic acid delivery in macrophage autophagy and immune reprogramming. Through combined transcriptomic and proteomic profiling identifying 1,363 differentially expressed genes and 384 proteins, they revealed convergent signaling pathways, including AMPK/mTOR and NF-κB, as central regulators of host responses. This work demonstrates how multi-omics analysis can link intracellular signaling, immune modulation, and translational delivery platforms within a unified conceptual framework, establishing exosomes as both mechanistic probes and therapeutic vehicles.

Comparative genomics provides complementary systems-level insights. Dubniewicz et al. reveal serovar-specific genomic features in *Leptospira interrogans* Hardjo associated with host adaptation, highlighting how genome analysis identifies determinants of host specificity and pathogenic behavior. Extending beyond sequence comparison, Lewicka et al. demonstrate that saccharide profiling refines pathogen classification by revealing structural similarities between antigenically related *Leptospira* strains indistinguishable by conventional serology, prompting re-evaluation of bacterial lipopolysaccharide evolution. The integration of genomic and glycomic data (Dubniewicz et al.; Lewicka et al.) illustrates how multi-platform approaches converge to improve pathogen classification, evolutionary insight, and translational relevance (Kwok et al., 2021; Geng et al., 2026).

Artificial intelligence and machine learning have revolutionized therapeutic target identification and validation (Pun et al., 2023). González-García et al. advance *Trypanosoma cruzi* N-myristoyltransferase as a drug target through large-scale *in silico* screening combined with experimental verification, demonstrating selective inhibition and biochemical tractability. This work establishes a reproducible framework for computationally driven target discovery in kinetoplastid parasites, enabling efficient navigation of chemical space while maintaining biological relevance (Chaudhary and Roos, 2005). Structural biology strengthens computational discovery by providing mechanistic context for therapeutic intervention. Tjia-Fleck et al. employ high-resolution cryo-electron microscopy to analyze membrane-bound streptococcal α-enolase in complex with human plasminogen, revealing specific interaction interfaces critical for host tissue invasion. These structural insights inform both target validation and rational inhibitor design, underscoring how computational and experimental methods reinforce one another (Childs-Disney et al., 2022).

The synergy between computational prediction and experimental validation extends to resistance mechanisms. Bhusal et al. comprehensively review drug resistance in leishmaniasis, synthesizing evidence showing how genomic plasticity, gene amplification, and regulatory rewiring collectively drive therapeutic failure. Their analysis of multi-layer data integration approaches highlights how computational modeling can distinguish adaptive resistance mechanisms from experimental noise, emphasizing resistance as a dynamic systems property (Dekker, 2024; Hossain et al., 2024).

Reproducibility in biomedical research necessitates integrated statistical frameworks from data acquisition through biological interpretation. Strzelecki and Nowicki provide a comprehensive review of tools for studying microbial iron homeostasis and oxidative stress, emphasizing standardized measurement, validation strategies, and cross-platform consistency. By highlighting both current methodological capabilities and critical gaps limiting reproducibility, this work reinforces that statistical rigor is not auxiliary but foundational for translating molecular findings into reliable therapeutic strategies. Methodological standardization proves equally critical in glycomics and proteomics. The saccharide profiling platform developed by Lewicka et al. demonstrates how analytical chemistry advances enable discrimination of closely related bacterial strains, providing orthogonal validation to genomic approaches. Similarly, the multi-omics integration framework employed by Zhu et al. showcases how rigorous statistical validation across transcriptomic and proteomic datasets strengthens mechanistic conclusions about immune reprogramming.

The intricate regulatory networks governing host-pathogen interactions involve dynamic interplay of metabolic, immunological, and evolutionary strategies. Shoeran et al. characterize SNF1 kinase homologs in *Leishmania major*, demonstrating how metabolic regulation contributes to parasite fitness, stress tolerance, and virulence. This work identifies regulatory kinases as promising intervention points while illustrating that targeting network regulators may offer more durable therapeutic strategies than single-target inhibition (Yu et al., 2022; Pang et al., 2022). Parasites showcase remarkable metabolic flexibility and adaptive responses to host-induced stress. The NF-κB pathway serves as a key host immune regulator that pathogens exploit for immune evasion, as demonstrated in the exosomal signaling studies by Zhu et al., while the AMPK/mTOR signaling axis links metabolic status with immune function. Autophagy plays a dual role, aiding pathogen degradation yet being manipulated by parasites for survival, a theme explored through multi-omics profiling of macrophage responses (Zhu et al.).

Translating laboratory discoveries to field applications requires integration of automation, miniaturization, environmental resilience, and data analytics (Conway et al., 2021). Álvarez-Rodríguez et al. develop a CRISPR-Cas-based recombinase polymerase amplification assay enabling ultra-sensitive detection of active *Trypanosoma brucei evansi* infections. This platform demonstrates how molecular innovation can be adapted for rapid, field-relevant diagnostics essential for surveillance and treatment decision-making in endemic settings. The isothermal amplification strategy eliminates the need for thermocycling equipment, enabling point-of-care deployment in resource-limited environments. Nano-enabled therapeutics represent another translational frontier. Sholkamy et al. advance sustainable zinc oxide nanoparticle synthesis from *Persicaria lapathifolia*, demonstrating antibacterial and anticancer activity while addressing scalability and environmental constraints. Combined with biological delivery systems such as exosome-mediated nucleic acid transport (Zhu et al.) and bionic camouflage (Rawat et al., 2025), these contributions illustrate how discovery-driven research can align with practical deployment considerations.

Structural validation through cryo-electron microscopy (Tjia-Fleck et al.) strengthens translational pipelines by providing atomic-resolution characterization of therapeutic targets. The computational target discovery framework (González-García et al.) accelerates lead optimization, while the diagnostic platform (Álvarez-Rodríguez et al.) enables real-time monitoring of therapeutic efficacy in clinical settings. Methodological standardization, as emphasized by Strzelecki and Nowicki, ensures that translational platforms maintain reproducibility across implementation sites. The glycomic profiling methods (Lewicka et al.) and genomic characterization approaches (Dubniewicz et al.) provide complementary diagnostic modalities for pathogen identification and epidemiological surveillance.

Several key themes emerge from this Research Topic: multi-omics integration reveals systems-level properties invisible to single-platform analyses (Zhu et al.; as reviewed by Bhusal et al.), computational methods accelerate target discovery when coupled with rigorous experimental validation (González-García et al., Tjia-Fleck et al.), statistical frameworks ensuring reproducibility are foundational rather than optional (Strzelecki and Nowicki), regulatory network analysis identifies intervention points offering therapeutic durability (Shoeran et al.), and translational considerations must be embedded from discovery inception rather than added retrospectively (Álvarez-Rodríguez et al., Sholkamy et al.). Looking forward, the framework established here provides a roadmap for addressing infectious disease complexity through coordinated, interdisciplinary research. As technologies advance from spatial multi-omics to AI-driven drug design, the principles of integration, validation, and translation exemplified in these works will remain essential. The convergence of computational power, experimental precision, and translational focus positions the field to make transformative progress against parasitic and infectious diseases that continue to challenge global health.

## References

[B1] Chaudhary K. Roos D. S. (2005). Protozoan genomics for drug discovery. Nat. Biotechnol. 23, 1089–1091. 10.1038/nbt0905-1089 16151400 PMC7096809

[B2] Childs-Disney J. L. Yang X. Gibaut Q. M. R. Tong Y. Batey R. T. Disney M. D. (2022). Targeting RNA structures with small molecules. Nat. Rev. Drug Discov. 21, 736–762. 10.1038/s41573-022-00521-4 35941229 PMC9360655

[B3] Clayton C. (2019). Regulation of gene expression in trypanosomatids: living with polycistronic transcription. Open Biol. 9, 190072. 10.1098/rsob.190072 31164043 PMC6597758

[B4] Conway L. P. Li W. Parker C. G. (2021). Chemoproteomic-enabled phenotypic screening. Cell Chem. Biol. 28, 371–393. 10.1016/j.chembiol.2021.01.012 33577749

[B5] Dekker J. P. (2024). Within-host evolution of bacterial pathogens in acute and chronic infection. Annu. Rev. Pathol. Mech. Dis. 19, 203–226. 10.1146/annurev-pathmechdis-051122-111408 37832940

[B6] Geng A. Cui C. Luo Z. Xu J. Meng Y. Cui F. (2026). Computational methods for spatial multi-omics integration. Biotechnol. Adv. 87, 108807. 10.1016/j.biotechadv.2026.108807 41564956

[B7] Hossain T. Singh A. Butzin N. C. (2024). Bacterial persisters: molecular mechanisms and therapeutic development. Signal Transduct. Target. Ther. 9, 170. 10.1038/s41392-024-01866-5 39013893 PMC11252167

[B8] Kwok A. J. Mentzer A. Knight J. C. (2021). Host genetics and infectious disease: new tools, insights and translational opportunities. Nat. Rev. Genet. 22, 137–153. 10.1038/s41576-020-00297-6 33277640 PMC7716795

[B9] Loiseau C. Cooper M. M. Doolan D. L. (2020). Deciphering host immunity to malaria using systems immunology. Immunol. Rev. 293, 115–143. 10.1111/imr.12814 31608461

[B10] Pang Y. Wu L. Tang C. Wang H. Wei Y. (2022). Autophagy-inflammation interplay during infection: balancing pathogen clearance and host inflammation. Front. Pharmacol. 13, 832750. 10.3389/fphar.2022.832750 35273506 PMC8902503

[B11] Pun F. W. Ozerov I. V. Zhavoronkov A. (2023). AI-powered therapeutic target discovery. Trends Pharmacol. Sci. 44, 561–572. 10.1016/j.tips.2023.04.001 37479540

[B12] Rawat P. Jahan F. Raman Suri C. Sahoo D. K. (2025). Bionic camouflage: integrating RBC membrane with biomimetic nanoparticles for enhanced immune evasion and targeted drug delivery. Mater. Today Bio 30, 101391. 10.1016/j.mtbio.2025.101391 39790487

[B13] Sholkamy E. N. Abdelhamid M. A. A. Rebai H. Sivapunniyam A. Ki M.-R. Pack S. P. (2025). Sustainable synthesis of zinc oxide nanoparticles from *persicaria Lapathifolia*: versatile anticancer and antibacterial applications. Front. Mol. Biosci. 12, 1601811. 10.3389/fmolb.2025.1601811 40692720 PMC12277169

[B14] Yu Y. Pan J. Liu M. Jiang H. Xiong J. Tao L. (2022). Guanylate-binding protein 2b regulates the AMPK/mTOR/ULK1 signaling pathway to induce autophagy during *Mycobacterium bovis* infection. Virulence 13, 875–889. 10.1080/21505594.2022.2075788 35531887 PMC9132469

